# Future Oceanic Warming and Acidification Alter Immune Response and Disease Status in a Commercial Shellfish Species, *Mytilus edulis* L.

**DOI:** 10.1371/journal.pone.0099712

**Published:** 2014-06-13

**Authors:** Clara L. Mackenzie, Sharon A. Lynch, Sarah C. Culloty, Shelagh K. Malham

**Affiliations:** 1 Centre for Marine Biodiversity and Biotechnology, School of Life Sciences, Heriot-Watt University, Edinburgh, United Kingdom; 2 Aquaculture and Fisheries Development Centre, School of Biological, Earth and Environmental Sciences, University College Cork, Cork, Ireland; 3 Centre for Applied Marine Sciences, Bangor University, Menai Bridge, Anglesey, United Kingdom; Fish Vet Group, Thailand

## Abstract

Increases in atmospheric carbon dioxide are leading to physical changes in marine environments including parallel decreases in ocean pH and increases in seawater temperature. This study examined the impacts of a six month exposure to combined decreased pH and increased temperature on the immune response and disease status in the blue mussel, *Mytilus edulis* L. Results provide the first confirmation that exposure to future acidification and warming conditions via aquarium-based simulation may have parallel implications for bivalve health. Collectively, the data suggests that temperature more than pH may be the key driver affecting immune response in *M. edulis*. Data also suggests that both increases in temperature and/or lowered pH conditions may lead to changes in parasite abundance and diversity, pathological conditions, and bacterial incidence in *M. edulis*. These results have implications for future management of shellfish under a predicted climate change scenario and future sustainability of shellfisheries. Examination of the combined effects of two stressors over an extended exposure period provides key preliminary data and thus, this work represents a unique and vital contribution to current research efforts towards a collective understanding of expected near-future impacts of climate change on marine environments.

## Introduction

Increases in atmospheric carbon dioxide are leading to physical changes in marine environments. Under the IPCC IS92a CO_2_ emission scenario, ocean pH is expected to decrease by 0.2–0.4 units by the year 2100 [1], [2]. In addition to changes in ocean pH, organisms will also have to contend with simultaneous increases in seawater temperature with the current average sea surface temperature of 19.7°C predicted to rise to 22.7°C by the end of the century [2], [3].

The ability of marine organisms to adjust to future climate change conditions will be critical to their health and ultimate survival. Bivalves are unable to thermo-regulate and are considered poor regulators of haemolymph acid-base balance [4], [5]. Consequently, pH and temperature changes in the environment will likely have direct influence on bivalve haemolymph pH and temperature. Additionally, any physiological mechanism associated with the bivalve circulatory system is liable to be affected under hypercapnic conditions. The bivalve immune system, for example, exists as an integrative part of the circulation system [6], [7]. The circulating cells, or haemocytes, represent the major cellular component of the animal's immune response and are responsible for a number of defence activities including phagocytosis or encapsulation of foreign or diseased cells, release of reactive oxygen metabolites and enzymes, and secretion of cytotoxic molecules [6], [8], [9], [10].

While prior investigations of the consequences of near-future changes in ocean pH and temperature have demonstrated a number of effects on marine organism physiology including impacts on growth, calcification and acid-base status [4], [11]–[18], less confirmed is how coinciding stressors might influence immune response in organisms [19]. Changes to single environmental factors, particularly temperature [20]–[24] but also salinity [25], pH [26] and dissolved oxygen [27] have demonstrated impacts on various immunological parameters including haemocyte numbers, phagocytosis and oxidative burst response.

The immune system is a major physiological mechanism ensuring host survival in the battle with pathogenic or parasitic organisms [28]. As stress impacts immune function and such mechanisms are the primary line of defence against pathogens, there is likely a strong link between immune response and the outbreak of disease in shellfish culture [29], [30]. Thus, climate change impacts on immunological aspects of physiology may be paralleled by associated changes in bivalve disease status.

Global climate change has increased pathogen development and survival, disease transmission and host susceptibility in the last thirty years [31]–[40]. Increased disease development in certain ecosystems can contribute to species decline and even extinction. Disease dynamics in the marine environment are being influenced by physical, chemical and biological alterations driven by climate change and such impacts will affect the sustainability of the shellfish industry. Multiple climate drivers such as ocean acidification and warming ocean temperatures have the potential to promote pathogen range expansion, infected host invasions and native host decline [41], [42] and tropical pathogen range contractions may also occur [43]. Additionally, it is possible that certain pathogens will be detrimentally affected by climate change conditions via changes to their required conditions thus allowing a relatively small number of emergent pathogens to infect new hosts with little or no tolerance [31].

Previous studies have shown associations between parasites and changing ocean temperatures, with a warmer environment being more favourable to the parasite [44]–[49]. Higher temperatures resulting in lower oxygen levels may stress organisms thus increasing their susceptibility to disease [50], [51], [52]. In the temperate zone, shorter milder winters are expected to increase the frequency and intensity of transmission of diseases [31]. Certain parasites also have higher growth rates, higher reproductive ability and decreased generation time with higher temperatures [53], [54].

Prior investigation also indicates that ocean acidification and associated stressors will be negative for calcifying organisms with more energy being required to build shells, which will reduce the amount of energy available to find food, reproduce or resist parasites and disease [26], [55], [56], [57]. It is possible that ocean acidification will also have a regulatory effect on certain parasite populations especially those with free-living developmental stages [58], [59]. However it is also possible that increased pathogenicity will occur if certain parasites are less affected by an acidified environment than their respective hosts [60].

This research aimed to examine the impacts of exposure to future ocean warming and acidification on the immune response and disease status of a commercially important bivalve species, *Mytilus edulis* (Linnaeus). The impacts of six months exposure to lowered pH and increased temperature on immunological responses, including haemocyte counts, phagocytosis, and nitroblue tetrazolium (NBT) reduction, and the health status of the mussels by histological and molecular examination were investigated.

## Materials and Methods

### Ethics statement

No specific permits were required for the study, which complied with all relevant regulations. The species collected in this study is not endangered or protected.

### Study organisms

*Mytilus edulis* (mean shell length  = 50.51 mm, SD  = 3.68 mm) were collected from a sub-tidal population in the Menai Strait, North Wales, UK on May 3^rd^, 2011 and acclimatised in large (200 L) flow-through holding tanks (12.5°C±SD 0.26°C, pH 8.01±SD 0.08, Salinity 34± SD 1 psu, 12L:12D light regime) at the School of Ocean Sciences, Bangor for a 3 week time period. During this time, mussels were drip-fed concentrated algal feed (Instant Algae Shellfish Diet 1800, Reed Mariculture, Campbell, CA, USA; 40% *Isochrysis* sp., 15% *Pavlov*a sp., 25% *Tetraselmis* sp. and 20% *Thalassiosira weissglogii* (Grunow); 52% protein, 16.1% lipid, 22.0% carbohydrate and 9.9% ash) at a ration of 27 mg dry mass mussel^−1^ day^−1^.

### Experimental set-up

Experimental set-up and conditions for this study are described in detail in Mackenzie *et al*. [61]. Briefly, *M. edulis* were exposed to current (∼400 µatm) and future (∼1000 µatm) *p*CO_2_ levels in an aquarium-based CO_2_ system. Current and future seawater conditions were simulated with seawater held at two temperatures (ambient, ambient +4°C) and two pH levels (ambient, ambient - 0.4 pH units) as predicted under the IPCC IS92a CO_2_ emission scenario [62]. This set-up allowed for examination of the interactive effects of varying pH and temperature conditions and included four treatments: ambient pH at ambient temperature (ambient), ambient pH at ambient temperature +4°C (warming), reduced pH at ambient temperature (acidified) and reduced pH at ambient temperature +4°C (acidified+warming).

Following input of seawater from the Menai Strait to an internal settling tank, sea water was filtered and UV treated before delivery to each of four header tanks (150 L) representative of the four experimental treatments. In-line heaters (Elecro Titanium Digital Heater) and cooling units (Aqua Medic TITAN 200) were used to maintain experimental temperatures. A pH controller (Walchem dual input pH controller) regulated addition of CO_2_ (g) to achieve acidified treatments.

Experimental header tanks gravity-fed (flow rate ∼5 ml second^−1^) seawater to 20 replicate 1.5 L tanks per treatment with overflows running to waste. Eight individual mussels were assigned to each tank per treatment. Mussels were acclimated to temperature treatments at an increase of 0.5°C day^−1^. Mussel tanks were cleaned three times per week and mussels were fed according to amounts and rates described for the acclimatisation period.

### Seawater parameters

Collection, storage and analyses of seawater carbonate samples are described in detail in Mackenzie *et al*. [61]. Briefly, 60 mL total alkalinity and dissolved inorganic carbon (DIC) seawater samples were collected fortnightly and sent to the Carbonate System Facility (LIMS) at the National Oceanography Centre, Southampton for total alkalinity and DIC analysis. Temperature (Mettler Toledo SG2 SevenGO, MT Ltd., Leicester) and salinity (TMC V2 ATC) of samples were also recorded at time of collection. Additionally, 30 mL nutrient samples were collected, filtered and frozen for analysis of phosphate and silicate concentrations at the Scottish Marine Institute. All values were entered into the CO2SYS model [63] to determine seawater carbonate parameters (i.e. pH, pCO_2_, HCO_3_, CO_3_^−2^, ΩAr, ΩCa) using the thermodynamic constants of Mehrbach *et al*. [64] refitted by Dickson and Millero [65]. Carbonate chemistry values for the four experimental treatments are provided in Table 1. Values represent the mean ±SD of bimonthly measures taken over the six months exposure period (n = 12 for each treatment).

**Table 1:** Seawater carbonate chemistry values for experimental treatments.

Treatment	T	Sal	TA	DIC	pH_T_	pCO_2_	HCO_3_^−^	CO_3_^−2^	Ωarag	Ωcalc
	(°C)	(‰)	(µmol/kg)	(µmol/kg)		(µatm)	(µmol/kg)	(µmol/kg)		
**Ambient**	12.14	33.58	2263.57	2093.78	7.99	465.82	1959.02	125.46	1.91	3.00
	(±0.48)	(±0.72)	(±15.70)	(±12.69)	(±0.05)	(±63.15)	(±30.99)	(±12.67)	(±0.20)	(±0.31)
**Warming**	15.84	33.71	2263.75	2087.47	7.95	516.77	1942.52	131.45	2.02	3.15
	(±0.27)	(±0.69)	(±15.99)	(±13.67)	(±0.03)	(±40.94)	(±18.28)	(±8.00)	(±0.12)	(±0.19)
**Acidified**	12.18	33.63	2264.69	2213.62	7.65	1087.69	2113.12	63.35	0.97	1.52
	(±0.48)	(±0.71)	(±16.97)	(±19.08)	(±0.06)	(±169.87)	(±21.27)	(±9.57)	(±0.14)	(±0.23)
**Acidifed+Warming**	16.11	33.54	2265.32	2205.95	7.63	1161.35	2101.03	69.07	1.06	1.66
	(±0.28)	(±0.72)	(±16.50)	(±15.69)	(±0.05)	(±136.86)	(±17.23)	(±7.37)	(±0.11)	(±0.17)

Standard deviations shown in parentheses (±SD) below corresponding mean value (n = 12). Measured values: temperature (T), salinity (Sal), total alkalinity (TA), and dissolved inorganic carbon (DIC). Modelled values: pH total scale (pH_T_), CO_2_ partial pressure (pCO_2_), bicarbonate (HCO_3_^−^), carbonate (CO_3_^−2^), aragonite saturation state (Ω_arag_) and calcite saturation state (Ω_calc_).

### Immunological response

#### Mussels

An initial group of adult *M. edulis* (n = 5) were sampled to determine baseline immune status. Following six months of exposure, five mussels were randomly selected from five experimental tanks (i.e. replicates) within each treatment (n = 5). Haemolymph samples of 0.3–0.6 mL were withdrawn from the sinus of the posterior adductor muscle of each mussel using 2 mL syringes and 25 G ½ needles. Five mussels were bled consecutively so that total haemocyte counts, phagocytosis counts, and NBT analysis could be carried out concurrently.

#### Total haemocyte counts

A 20 µL haemolymph subsample from each mussel was used to determine total haemocytes ml^−1^, counted using an improved Neubauer haemocytometer [7].

#### Phagocytosis

Phagocytosis assay was performed as previously described [29], [66] with some modifications. Briefly, a 20 µL subsample of haemolymph was placed onto a glass slide and allowed to adhere for 20 min in a moist incubation chamber before the addition of 20 µL of fluorescein 5-isothiocyanate Isomer 1 (FITC) labelled *E. coli* (Sigma). Glass slides were then incubated for a further 20 min after which all were rinsed with Tris-HCl buffer solution containing 2% NaCl (pH 7.6). Unphagocytosed bacteria were counterstained with ethidium bromide (Sigma) and then rinsed clear with Tris-HCL. Triplicate counts of 200 cells were immediately carried out using a 488 nm emission filter on a Zeiss microscope.

#### Nitroblue tetrazolium (NBT) reduction

A modified version of the nitroblue tetrazolium (NBT) assay, as described by Pipe [67] and Bussell *et al*. [25] was applied. Twenty µL of haemocyte suspension were added to 96 well plates (Fisherbrand) in triplicate for each sample. Negative and positive controls were incorporated. Following incubation for 20 min, 20 µL of nitroblue tetrazolium (NBT) (Sigma) (2 mg/ml in Tris–HCl buffer containing 2% NaCl (pH 7.6)) were added to one set of triplicate wells for each sample. Twenty µL Tris buffer were added to corresponding negative control wells. The well plates were then incubated for a further 20 min before centrifugation (1000 g at 10°C for 10 min). All wells were then washed with Tris-HCl buffer solution before another centrifugation (x2) (1000 g) after which 20 uL of methanol (50%) were added before a final centrifugation. The plates were air dried before the addition of 240 uL potassium hydroxide (KOH) and 280 uL dimethylsulphoxide (DMSO) to all plate wells. Optical density (OD) values were measured on a Dynex MRX-II spectrophotometer (λ = 620 nm).

### Disease status

#### Mussels

An initial sample of adult *M. edulis* (n = 30) was screened from the Menai Strait in May 2011 to determine the health status of the mussels in the field. Following six months of exposure, twelve mussels were randomly selected from twelve experimental tanks (i.e. replicates) within each treatment for determination of health status by histological and molecular examination (n = 12).

#### DNA extraction

DNA was extracted from the initial mussel sample (n = 30) (gill tissue stored in 95% ethanol) using 10% Chelex 100 resin [68], [69]. DNA was extracted from the corresponding paraffin-embedded tissue of each mussel from the laboratory trial consisting of twelve mussels per treatment (n = 12). Deparaffinization of the tissue was carried out [70] and DNA extractions were undertaken using protein precipitate and cell lysis using the QIAamp DNA Mini Kit (Qiagen).

#### Standard polymerase chain reaction (PCR)

Several PCRs were carried out to determine (1) which Mytilus species was being screened, (2) general bacterial screening, (3) general microbial screening, (4) presence or absence of Marteilia refringens (Grizel et al) and general haplosporidian spp. screening.

(1) PCR for species identification: Initially a PCR was carried out to detect the nuclear DNA markers Me15/Me16 [71] to confirm that *M. edulis* was being screened as hybrid zones consisting of *M. edulis*, *Mytilus galloprovincialis* (Lamarck) and hybrids of both parent species are known to occur on the southwest coast of the UK. The PCR mastermix was modified slightly to include 5× green buffer. Amplification was conducted in 25 µl of the reaction mixture containing 14 µl ddH_2_O, 5 µl 5× green buffer (Promega), 2.5 µl of each of the four deoxyribonucleotide triphosphates dNTPs (dATP, dCTP, dGTP, dTTP) (0.2 mM), 1.5 µl MgCl_2_ (25 mM stock), 0.5 µl of the primer Me15 (5′-CCAGTATACAAACCTGTGAAGA-3′), 0.5 µl of the primer Me16 (5′-TGTTGTCTTAATGGTTTGTAAGA-3′) (100 pmol/µL stock), 1 µl of Go Taq DNA polymerase (0.03 U) and 1 µl of total DNA. Negative controls consisting of ddH_2_0 were used. The following conditions were used for the PCR in a thermocycler: 94°C for 30 s, 55°C for 45 s and 70°C for 90 s (40 cycles). The expected product size for *M. galloprovincialis* is 126 bp while in *M. edulis* it is 180 bp. In hybrids both bands occur simultaneously at 126 bp and 180 bp.

(2) Bacterial screening: PCR was carried out to screen for general bacterial species that might be present using the universal primers EUBB and EUBA [72], which amplify the entire 16srRNA region. The PCR mastermix was modified to include 5 µl 5× green buffer, 5 µl dNTP (0.2 mM), 0.5 µl of MgCl_2_ (25 mM stock), 0.25 µl of each primer (EUBB and EUBA) (100 pmol/µL stock) and 0.1 µl GoTaq DNA polymerase (0.03 U) per PCR reaction. The PCR reaction mix was made up to a volume of 20 µl using ddH20 and 5 µl of undiluted genomic DNA. The following conditions were used for the PCR in a thermocycler: 95°C for 5 min, 35 cycles of 95°C for 30 s, 55°C for 30 s and 72°C for 30 s with a final extension at 72°C for 10 min. The expected product size is 1.5 Kbp. A second pair of primers, UNIV16s EUB f933/UNIV16s EUB r 1387 [73] which are specific for universally conserved bacterial 16s rDNA region were used for the bacterial screening. The PCR mastermix and thermocycling conditions were modified. The PCR mastermix was made up to a volume of 49 µl for each PCR reaction: 37.75 µl ddH_2_0, 10 µl 5× green buffer, 1 µl dNTPs (0.2 mM), 3 µl MgCl_2_ (25 mM stock), 1 µl of each primer (100 pmol/µL stock) and 0.25 µl GoTaq DNA polymerase (0.03 U). 1 µl of undiluted genomic DNA was screened. The PCR was carried out in a thermocycler as follows: 95°C for 1 min, 35 cycles at 94°C for 20 s, 56°C for 30 s, 72°C for 30 s and a final extension at 72°C for 7 min.

(3) Microbial screening: Universal primers 18ScomF1/18ScomR1 [74], which amplify the 18Scom region, were used for a general health screen to assess if any other foreign DNA in mussel tissue could be detected.

(4) Screening for potential mussel parasites: A PCR protocol targeting the ITS1 was carried out to detect *M. refringens* [75]. Several generic haplosporidian PCRs were carried out using the HAP-F1 and HAP-R3 primers [76] and the ssu980 and HAP-R1 primers [76], [77]. Electrophoresis of the amplification products from all PCRs was conducted in a 2% agarose gel. Fifteen µL of ethidium bromide, EtBr, (10 mg/ml stock) was added to the agarose gel and the gel was run at 110 V for 60 min.

#### Sequencing

Direct sequencing was carried out on products amplified by eurofins MWG using ABI 3730xl 96-capillary DNA Analyzers. DNA from pooled PCR products (n = 3/n = 4) from multiple individuals in each sample were isolated and purified using the Qiaquick gel extraction kit (Qiagen) prior to direct sequencing Both the forward and reverse strands of DNA samples were sequenced commercially (MWG eurofins). Each sequence was matched against the National Centre for Biotechnology Information (NCBI) nucleotide database with BLASTn (Basic Local Alignment Search Tool) to identify the species present.

#### Histology

A transverse section of mussel tissue (∼1 cm^2^) containing mantle, gill, digestive gland and gonad tissue was excised and fixed in 96% ethanol initially and tissue sections were subsequently transferred to Davidson's fixative at 4°C for 48 hrs [78] before being processed. The fixed tissue was dehydrated through an ascending ethanol series and embedded in paraffin. 5 µm tissue sections were stained using haematoxylin and eosin. Tissue slides were scanned for any pathological or morphological observations and macro- or microparasites at a magnification of 40× and under oil at 100x. The location and intensity of each type of parasite were recorded and the prevalence and intensity of infection were calculated where possible.

### Statistical analyses

All statistical analyses were carried out in SPSS 14.0 for Windows (2005). Two-way ANOVAs were applied to determine the effect of pH, temperature and their interaction on immunological parameters (p<0.05). Data was tested for normality and homogeneity of variances (Levene's Test). One-way ANOVA were used to directly compare treatments. If necessary, data was log-transformed to meet assumptions. Significant difference in the diversity and prevalence of each parasite group and pathological condition, with varying pH and temperature, was calculated using chi-square (χ^2^) analysis with significance determined at p<0.05.

## Results

### Immunological response

#### Total haemocyte counts

**Figure 1:**
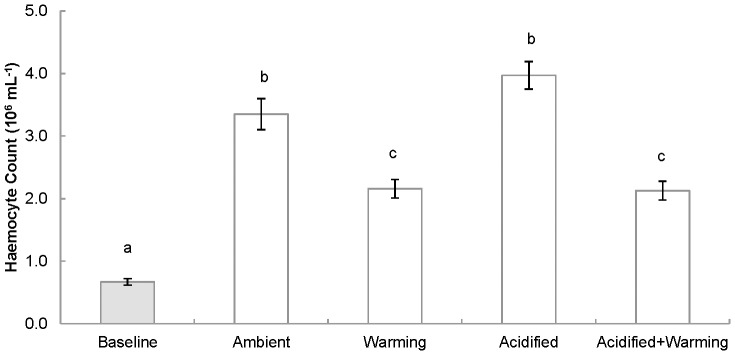
Effects of temperature and pH on haemocyte count (10^6^ mL^−1^) (±1 SE) in *Mytilus edulis* (n = 5) following a six month exposure period to varying temperature and pH conditions. Baseline value shown in grey scale. Lowercase letters indicate significant differences.

Two-way ANOVA identified a significant negative effect of increased temperature on haemocyte counts but neither an influence of pH nor interaction between pH and temperature (Table 2). Haemocyte counts varied significantly between groups (F = 24.336; p<0.001) with *post-hoc* tests revealing that baseline animals had significantly lower haemocyte counts than any experimental treatments (ambient (p<0.001), warming (p = 0.001), acidified (p<0.001), acidified+warming (p = 0.001)). Animals held at either ambient temperature treatment (ambient, acidified) had significantly higher haemocyte counts than those under increased temperature treatments at either pH (warming (p = 0.005, p<0.001, respectively), acidified+warming (p = 0.004, p<0.001, respectively)) (Fig. 1).

**Table 2:** Two-way ANOVA results comparing the effects of pH and temperature on immunology (a. haemocyte counts, b. phagocytosis, c. NBT) in *Mytilus edulis* following a 6 month exposure period.

Source of variation	df	SS	MS	*F*-ratio	*P*-value
**a. Haemocyte Counts**					
pH	1	0.435	0.435	0.923	0.351
Temperature	1	11.476	11.476	24.343	**<0.001**
pH*Temperature	1	0.528	0.528	1.120	0.306
**b. Phagocytosis**					
pH	1	30.854	30.854	0.271	0.610
Temperature	1	1720.224	1720.224	15.133	**0.001**
pH*Temperature	1	649.686	649.686	5.715	**0.030**
**c. NBT**					
pH	1	0.186	0.186	4.740	**0.045**
Temperature	1	0.419	0.419	10.668	**0.005**
pH*Temperature	1	0.045	0.045	1.137	0.302

Significant values shown in bold (p<0.05).

#### Phagocytosis

**Figure 2:**
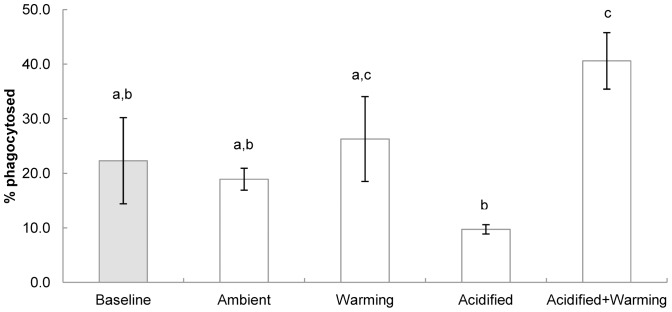
Effects of temperature and pH on phagocytosis (% phagocytosed haemocytes) (±1 SE) in haemocytes from *Mytilus edulis* (n = 5) following a six month exposure period to varying temperature and pH conditions. Baseline value shown in grey scale. Lowercase letters indicate significant differences.

Two-way ANOVA showed a significant positive effect of increased temperature on percentage of phagocytosed cells as well as a significant interaction between pH and temperature but no effect of pH (Table 2). The percentage of phagocytosed cells varied significantly between all groups (F = 3.846; p = 0.020) but failed assumptions of homogeneity of variances even after transformed. *Post-hoc* tests identified a significantly higher percentage of phagocytosed cells in haemolymph of mussels held at decreased pH and warming conditions (acidified+warming) than in baseline animals (p = 0.047) or at ambient temperature treatments at either pH (ambient (p = 0.016), acidified (p = 0.001)). Additionally, animals held at increased temperature and ambient pH (warming) had a significantly higher percentage of phagocytosed cell than those under ambient temperature and decreased pH (acidified) (p = 0.044) (Fig. 2).

#### Nitroblue tetrazolium (NBT) reduction

**Figure 3:**
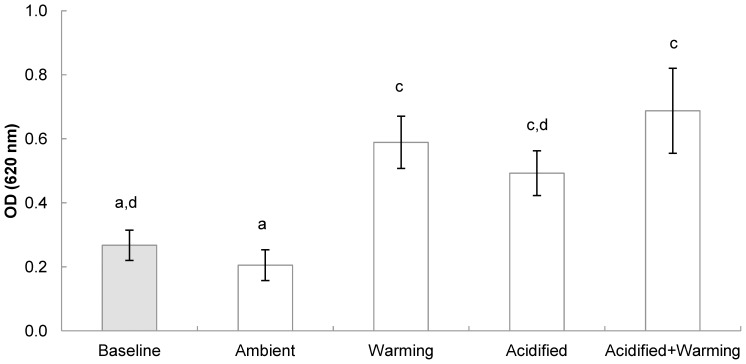
Effect of temperature and pH on the reduction of Nitroblue tetrazolium (NBT) (±1 SE) in haemocytes from *Mytilus edulis* (n = 5) following a six month exposure period to varying temperature and pH conditions (OD  =  optical density at λ = 620 nm). Baseline value shown in grey scale. Lowercase letters indicate significant differences.

Two-way ANOVA identified a significant positive effect of both decreased pH and increased temperature on NBT reduction but no interaction between the two factors (Table 2). NBT reduction varied significantly between treatments (F = 6.349; p = 0.002) with *post-hoc* tests revealing that mussels reared under ambient temperature and pH conditions (ambient) had significantly lowered reduction than all other experimental treatments (warming (p = 0.004), acidified (p = 0.022), acidified+warming (p<0.001)) Animals held under warming conditions at either pH (warming, acidified+warming) had significantly increased reduction than baseline animals (p = 0.012 and p = 0.002, respectively) (Fig. 3).

### Disease status

#### PCR and direct sequencing

(1) A product of 180 bp was amplified in the Me15/Me16 PCR confirming the mussel species to be *M. edulis* (Table 3). Direct sequencing confirmed the mussel species to be *M. edulis*.

**Table 3:** PCR screening results of the Menai Strait *Mytilus edulis* using different primer pairs.

Treatment	Me15	EUBA	Univ16s EUBf	18ScomF1	ITs	HAP-F1	ssu980
	Me16	EUBB	Univ16s EUBr	18ScomR1		HAP-R3	HAP-R1
**Initial Sample**	*Mytilus edulis*	0%	92%	7%	0%	60%	3%
			(28/30)	(2/30)		(18/30)	(1/30)
**Ambient**	*Mytilus edulis*	0%	92%	67%	0%	0%	0%
			(11/12)	(8/12)			
**Warming**	*Mytilus edulis*	33%	67%	25%	0%	0%	0%
		(4/12)	(8/12)	(3/12)			
**Acidified**	*Mytilus edulis*	0%	83%	0%	0%	0%	0%
			(10/12)				
**Acidified+Warming**	*Mytilus edulis*	0%	92%	0%	0%	0%	0%
			(11/12)				

(2) In the PCR using the universal primers for bacteria screening, EUBB and EUBA [72], multiple products were amplified at 800 bp, 900 bp and 1000 bp. The second PCR used to screen for bacteria using the Univ16s EUBf and Univ16s EUBr primers amplified a product at ∼500 bp (Table 3). Several bacterial species were detected in the initial sample and in the mussels from all four treatments. The highest prevalence was observed in mussels from the initial sample (92%), ambient treatment (92%) and acidified+warming treatment (92%) followed by the acidified treatment (83%) and warming treatment (67%). In the bacterial screening, no DNA sequences were recovered for the products amplified in the EUBA and EUBB PCR. Five forward and five reverse sequences were obtained from the products amplified in the Univ16sEUBf and Univ16s EUBr PCR from a subsample of infected individuals (n = 6). Bacterial species detected included a Gammaproteobacterium sp. (99% Query coverage, 98% Maximum identity), *Pseudomonas* sp. (98% Query coverage, 95% Maximum identity), *Pectobacterium carotovorium* (Jones) (100% Query coverage, 97% Maximum identity), *Xanthomonadaceae* sp. (99% Query coverage, 98% Maximum identity) and *Serratia* sp. (99% Query coverage, 97% Maximum identity).

(3) A product (∼350 bp) was amplified in the 18Scom region using the Zhang *et al*. [74] modified PCR. This product was amplified in mussels from the initial sample, warming and ambient treatments but was absent in mussels from the acidified and acidified+warming treatments. The free-living amoebae *Hartmannella vermiformis* (Page) (85% Query coverage, 99% Maximum identity) was identified using the 18Scom PCR.

(4) No products were amplified in the ITS region using the *M. refringens* PCR (Table 3). A product was amplified (350 bp) using the generic haplosporidian primers [76] in eighteen individuals in the initial sample (60% prevalence), however, no products were amplified in mussels from any of the experimental treatments. Direct sequencing of the products to confirm haplosporidian identification in the initial sample using the generic haplosporidian primers was unsuccessful. Using the second pair of haplosporidian primers (ssu980 and HAP-R1), a product was amplified at 430 bp in a single individual in the initial sample, which also amplified a product using the HAP-F1 and HAP-R3 primers. No products were amplified in the mussels from the experimental treatments (Table 3). In the direct sequencing, two forward and two reverse DNA sequences were obtained from the replicate PCR products amplified by the single mussel in the ssu980 and Hap-R1 PCR [76], [77]. Blastn analysis of the DNA isolated from *M. edulis* confirmed the DNA to be that from a previously undescribed haplosporidian (Accession no. KC852876 and KC852877) most similar to *Minchinia chitonis* (Lankester) (Accession no. AY449711.1, 95–100% Query coverage, 92–93% Maximum identity) and a haplosporidian of the Florida marsh clam *Cyrenoida floridana* (Dall) (Accession no. AY449712.1, 90–98% Query coverage, 93–95% Maximum identity). A phylogenetic tree was generated based on the majority rule jackknife consensus parsimony analysis with 1000 pseudo replicates with 75% character deletion using a heuristic search with 1000 random sequence additions each.

#### Histology

In total, seven parasite groups were detected in *M. edulis*. Haplosporidian like organisms (HLOs) were observed in the tissues of ten mussels (33% prevalence) from the initial sample (Table 4) but were not observed or detected in the final sample of mussels screened from any treatment group of the laboratory trial. The HLOs consisted of a cluster of cells of varying sizes (approximately 10 to 30 microns) each with an eccentric nucleus.

**Table 4:** Prevalence of parasites in *Mytilus edulis* by histology.

Treatment	Ciliate	*Mytilicola intestinalis*	Trematode	*Hartmannella* sp.	*Nematopsis* sp.	Prokaryote inclusions	*Marteilia refringens*	Haplosporidian sp.
**Initial sample**	6%)	27%	22%	50%	6%	0%	0%	33%
	(2/30	(8/30)	(6/30)	(15/30)	(2/30)			(10/30)
**Ambient**	0%	33%	0%	42%	0%	58%	0%	0%
		(4/12)		(5/12)		(7/12)		
**Warming**	25%	0%	25%	0%	0%	0%	0%	0%
	(3/12)		(3/12)					
**Acidified**	0%	33%	0%	33%	8%	83%	0%	0%
		(4/12)		(4/12)	(1/12)	(10/12)		
**Acidified+Warming**	0%	42%	8%	8%	0%	0%	0%	0%
		(5/12)	(1/12)	(1/12)				

Initial prevalence and prevalence following 6 month exposure to ambient, warming, acidified or acidified+warming seawater conditions are presented.

No single parasite species was detected across the initial sample and all four mussel treatment groups. However, the copepod *Mytilicola intestinalis* (Steuer) and the amoeba *Hartmannella sp*. were observed in the initial sample and three of the experimental treatment groups, being absent only in mussels from the warming treatment. The highest parasite diversity was observed in the initial sample (6 species) followed by the acidified treatment (4 species), the ambient and acidified+warming treatments (3 species) and warming treatment (2 species). A significant decrease in parasite diversity from the initial sample was observed in three of the experimental treatments (ambient (p = 0.046), warming (p = 0.014), acidified+warming (p = 0.046). All other diversity comparisons were not significant.

Most prevalence comparisons were significantly different for each parasite and treatment, with the parasite being either absent or the prevalence increasing or decreasing compared to the initial sample. However, some comparisons did have a similar prevalence. Comparing parasite prevalence within each treatment, in the initial sample most comparisons were significantly different except for ciliates and *Nematopsis* sp. (p = 1) and *M. intestinalis* and trematodes (p = 0.411). In the warming treatment, a similar prevalence was observed for ciliates and trematodes, the only parasite groups present (p = 1). Of the three parasite groups present in the ambient treatment, a significant difference in prevalence was observed between *M. intestinalis* and prokaryote inclusions (p = 0.0003) and *Hartmannella* sp. and prokaryote inclusions (p = 0.024). No significant difference was observed between *M. intestinalis* and *Hartmannella* sp. (p = 0.189). In the four parasite groups present in the acidified treatment mussels, most prevalence comparisons were significantly different (p<0.0001) except for *M. intestinalis* and *Hartmannella* sp. (p = 1). In the acidified+warming treatment, no significant difference (p = 1) was observed between trematodes and *Hartmannell*a sp.

**Figure 4:**
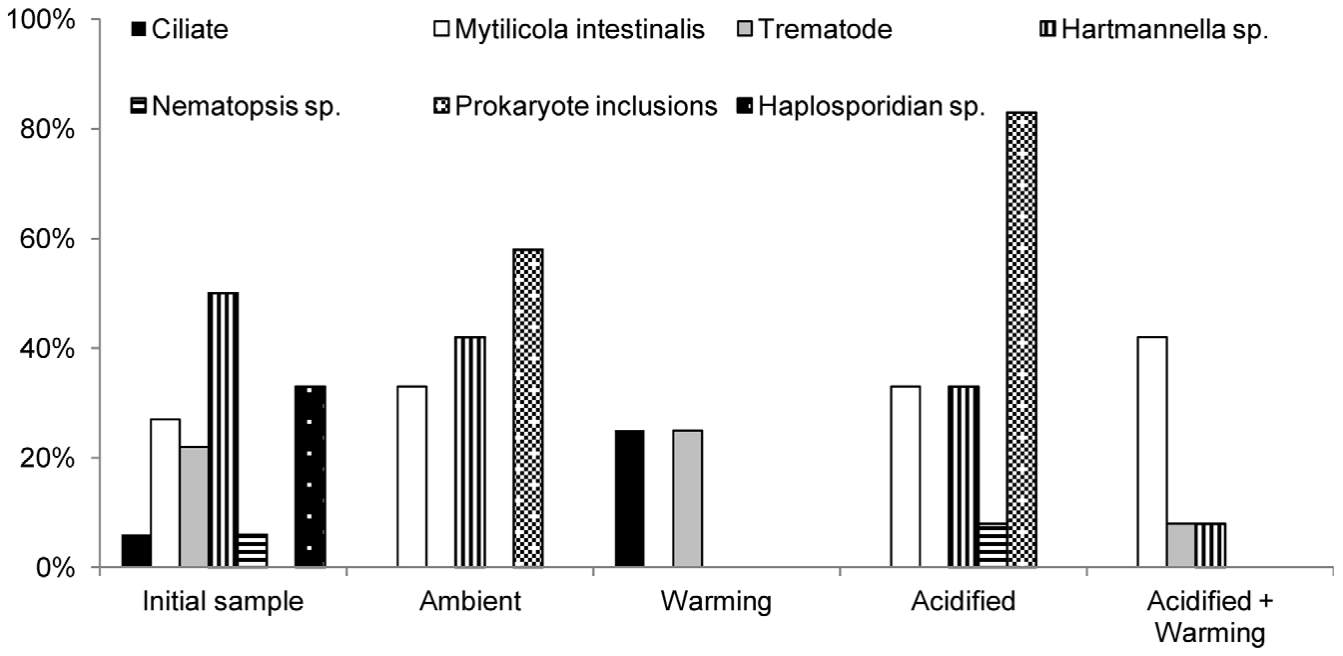
Prevalence of parasites in *Mytilus edulis*.

Ciliates were only present in mussels from the initial sample and the warming treatment, with prevalence quadrupled in the latter showing a significant difference in prevalence between both samples (p = 0.0002). An increase in the prevalence of *M. intestinalis* was observed in three of the four treatments (ambient, acidified, acidified+warming,) compared to the initial sample, with the prevalence increasing significantly (p = 0.026) in the acidified+warming treatment but was not significantly different in the ambient and acidified treatments (p = 0.3). A slight increase in the prevalence of trematodes from the initial sample was observed in the warming treatment, which was not significant (p = 0.6), whereas a significant decrease (p = 0.0056) in trematode prevalence was observed in the only other treatment (acidified+warming) in which trematodes were observed. Across all treatments, *Nematopsis* sp. was only present in one individual of the acidified treatment. Prokaryote inclusions were observed in a high proportion of mussels from the ambient and acidified treatments (58% and 83%, respectively), with a significant difference being observed between both treatments (p = 0.0001) (Table 4, Fig. 4).

#### Pathological conditions

**Figure 5:**
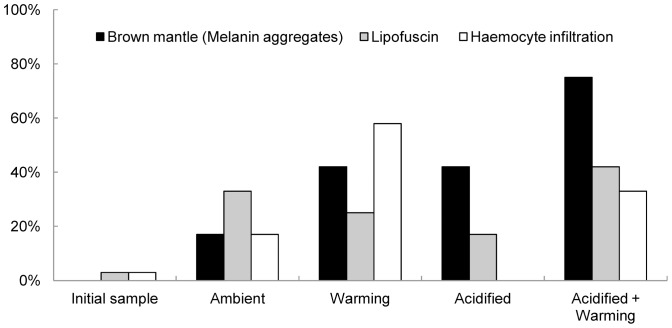
Prevalence of morphological changes in *Mytilus edulis*.

In total, three pathological conditions were observed in the *M. edulis*. These conditions included melanin aggregates in the mantle tissue, lipofuscin within the kidney epithelium [79] and focal haemocyte infiltration in the vascular connective tissue (Table 5, Fig. 5). All three conditions were observed in the four treatments however, melanin aggregates were not observed in the initial sample nor haemocyte infiltration in the acidified treatment. Mussels with the highest prevalence of melanin aggregates and lipofuscin, within the kidney epithelium, were observed in the acidified+warming treatment while the highest prevalence of haemocyte infiltration was observed in the warming treatment. No significant difference was calculated for the number of pathological conditions observed between samples.

**Table 5:** Prevalence of morphological changes in *Mytilus edulis* by histology.

Treatment	Brown mantle	Lipofuscin	Haemocyte infiltration
	(Melanin aggregates)		
**Initial sample**	0%	3%	3%
		(1/30)	(1/30)
**Ambient**	17%	33%	17%
	(2/12)	(4/12)	(2/12)
**Warming**	42%	25%	58%
	(5/12)	(3/12)	(7/12)
**Acidified**	42%	17%	0%
	(5/12)	(2/12)	
**Acidified+Warming**	75%	42%	33%
	(9/12)	(5/12)	(4/12)

Initial prevalence and prevalence following 6 month exposure to ambient, warming, acidified or acidified+warming seawater conditions are presented.

## Discussion

This is the first confirmation that coinciding exposure to future acidification and warming conditions has parallel implications for immune and disease status in *M. edulis*. Collectively, the data suggests that temperature more than pH may be the key driver affecting immune response in *M. edulis*. Haemocyte counts were lower under 16°C temperature conditions and phagocytosis activity increased under such conditions. Conversely, NBT reduction increased under 16°C conditions and/or pH 7.68 conditions, and therefore the effect of pH to immune function should not be discounted. Likewise, data suggests that both increases in temperature and/or lowered pH conditions may lead to changes in parasite diversity and prevalence, pathological conditions, and bacterial incidence in *M. edulis*. These changes may result in the removal or emergence of a parasite and an increase or decrease in parasite prevalence. No significant parasites such as *M. refringens* for example, were detected in this study, however, the detection of a previously undescribed HLO in the initial sample and its impact on that mussel stock, which has yet to be determined, is significant.

Previous research has shown that increased stress increases total haemocyte counts (THC) in bivalves [23], [80], [81]. However, investigation into the specific effect of temperature stress on THC has demonstrated both decreases [82], [83], [84] and increases [23] in marine bivalves. Our results suggest that temperature negatively influences THC as warming resulted in a decrease in circulating haemocytes.

Any metabolic adaptations to warming and/or acidification stressors could result in a transfer of energy resources to other physiological systems [85], potentially at the expense of maintaining haemocyte abundance. Therefore, the observed decline in THC may be indicative of an overall reduced energy state of *M. edulis* under stress conditions as been seen previously in marine bivalves under stress conditions [86]. Similarly, such declines may be representative of an overall depletion of energy reserves due to the extended exposure period (which included the species' natural spawning windows). Any immunological response mounted by the species to deal with stressors may also incur energetic costs including altered energy metabolism, altered fatty acid composition and reduced reproductive investment [86] and thus, an animal that has to sustain such a response over time will inevitably incur reduced energy stores. These in turn may lead to a decreased ability to mount an effective immunological response particularly if parallel shifts in pathology and parasite loads coincide. Consistent increases in THC in all treatments from baseline values could be a result of either proliferation of cells, or transfer of cells from tissues into circulation [8] in response to stress conditions. Acidification conditions did not appear to affect THC. Examination of the sole effect of acidified seawater conditions by Bibby *et al*. [26] also found no effect on THC.

Phagocytosis is considered a good measure of immune competency in bivalves [87]. Previous studies have demonstrated that phagocytic activity of haemocytes is affected by changes in both temperature [28] and acidification [26]. Stress conditions predominantly cause decreases in phagocytic activity [23], [26], [30], [88], Conversely, the current study observed significant increased phagocytosis in *M. edulis* under increased temperature with potentially some effect of lowered pH. Such changes were paralleled by an increase in oxidative burst (NBT). Reduced oxidative burst has been shown to decline in other bivalve species under conditions of temperature stress [20]. Increases in both immunological parameters may be indications of the general decline in condition of the animal and could also represent a higher “immunological load” as presented by changing pathogen and parasite condition induced by temperature and/or pH stressors.

A reduction in parasite diversity in the treatments compared to the initial sample may have been due to the artificial holding conditions in the laboratory or it may have been due to the altered pH, temperature and ecology of the parasite, such as the removal of other hosts. The HLOs observed in the initial sample were not observed in the mussels from the different treatments at the end of the trial. This is likely due to the removal of mortalities during the trial, which may have been been infected with the HLOs, while the survivors were uninfected giving a negative result in the final sample at the end of the trial.

Prokaryote inclusion bodies, which are bacterial cellular reserve material used during periods of special growth phases or under special environmental conditions [89], were not detected in the initial sample but were observed in both treatments held at 12°C and were most prevalent in the reduced pH treatment. A high prevalence of bacteria detected using PCR in all treatment samples would be expected due to the laboratory holding conditions.

The prevalence of *M. intestinalis*, which causes ‘red worm disease’, almost doubled in the acidified+warming treatment, when compared to the initial sample. Increasing temperature has been shown to be a factor influencing infestation rates [90], however, *M. intestinalis* was not detected in mussels from the warming treatment in this study but was present in all other three treatments which might indicate that this copepod prefers an environment with a reduced pH when seawater temperatures are elevated. *Hartmannella* sp., a free-living amoeba, was observed in most samples but was absent (warming) or significantly reduced (acidified+warming) in warming treatments indicating that this species may be sensitive to elevated seawater temperatures. Ciliates were only detected in the warming treatment but were absent in the acidified+warming treatment, which might indicate that they are sensitive to a reduced pH environment.

Trematodes were only observed in the two treatments held at 16°C, however, a prevalence reduction of almost a third was observed in the acidified-warming treatment. These results may indicate that the trematodes are sensitive to a more acidic and cooler environment, due to their absence in treatments held at 12°C.

In this study, all pathological conditions observed in the initial sample increased significantly in the four treatments, with an additional condition (melanin aggregates), which is part of a general inflammatory-like response in invertebrates [91], being observed in the laboratory trial mussels. Lipofuscin, lipid-containing residues of lysosomal digestion, is considered one of the ageing or “wear and tear” pigments and may be symptomatic of membrane damage or damage to mitochondria and is thus associated with the break down and absorption of damaged blood cells [92], [93]. In this study, all mussel samples had lipofuscin within the kidney epithelium with the acidified+warming treatment having the highest prevalence. Haemocyte infiltration had a significantly higher prevalence in mussels in the warming treatment but was absent in mussels in the acidified treatment. A positive correlation between haemocyte number and increasing seawater temperature has been recorded in the Mediterranean mussel, *M. galloprovincialis* [94] and in clams, *Ruditapes philippinarum* (Adams & Reeve) [95]. Monari *et al*. [23] suggested that the increased number of haemocytes found in the clam *Chamelea gallina* (Linnaeus) under elevated seawater temperatures, might have been a consequence of a mobilisation of cells from tissues to haemolymph, in order to respond to bacteria.

Intrinsic factors such as host immunity and pathogen virulence will be more difficult to predict in climate change studies [96]. Knowledge of the rate of pathogen evolution and host evolutionary response is critical in predicting disease spread and subsequent effects on biodiversity [31], [97]. Certain climate change driven stressors may also have a more negative impact on the parasite than on hosts [98]. Consequently, it is apparent that climate change-disease interactions are complex [38], [43], [96], [99], [100] and that an interdisciplinary approach should be taken [101].

## Conclusions

Ocean warming and acidification pose substantial threat to marine bivalves and similar thermo-conforming species with limited ability to regulate haemolymph pH. The immune system of marine bivalves such as *M. edulis* may become comprised under such conditions. Furthermore, the reduced immune capacity of *M. edulis* to cope with climate-induced stressors such as ocean warming and acidification while dealing with co-occurring changes in parasite loads and pathogen incidence poses advanced threat to the health of the species. As such, parallel impacts to the immune status and pathology of *M. edulis* could have serious implications for global fisheries that subsist on the production and export of this commercially valuable species [102]. In addition, any ecosystem services provided by *M. edulis* (e.g. uptake and recycling of energy and nutrients, bioturbation and bioirrigation of marine sediments, sediment/shoreline stabilization and habitat formation [103]) could also be threatened. We therefore highlight the need for further investigation into the long-term effects of collective climate-induced stressors to combined aspects of bivalve health and physiology so as to provide a comprehensive illustration of impacts, which could inform management and protection of *M. edulis* as well as other marine species of high global ecological and economic value.
